# Supplementary material for: Integrative Multiomics Profiling Unveils the Protective Function of Ulinastatin against Dextran Sulfate Sodium-Induced Colitis
Source: Antioxidants (Basel). 2024 Feb 8;13(2):214. doi: 10.3390/antiox13020214 (PMC10886110; doi:10.3390/antiox13020214)
Supplement: Supplementary file 1 [file antioxidants-13-00214-s001.zip › Supplementary Table S1.pdf]

Supplementary Table S1. Primer sequences.

Gene	Accession number	Forward primer	Reverse primer
Gapdh	NC_000072	AGGTCGGTGTGAACGGATTTG	TGTAGACCATGTAGTTGAGGTCA
Osm	NC_000077	CAGAATCAGGCGAACCTCACG	AGCTCTCAGGTCAGGTGTGTT
Csf3	NC_000077	ATGGCTCAACTTTCTGCCCAG	CTGACAGTGACCAGGGGAAC
Bcl2l1	NC_000068	GACAAGGAGATGCAGGTATTG G	TCCCGTAGAGATCCACAAAAGT
Gm13305	NC_000070	TCTGGGTTAGGACACAGACTG	GCTTGGCAAGAGACTTCAGGA
Il11ra1	NC_000070	AGCACTGGAGGGAGCAGA	TCCATACTGGACCCCTGGAG
Il11ra2	NC_000070	CTCTTGCCAAGCGGTAGACTA	GGATGGACTTTCCCTCTGACTC
Il3ra	NC_000080	CTGGCATCCCACTCTTCAGAT	GGTCCCAGCTCAGTGTGTA
Prl6a1	NC_000079	TGGAGGATGTGCGATATAAACC A	GGCAGGCAGTAAGGTATCTGT
Il22	NC_000076	ATGAGTTTTTCCCTTATGGGGA C	GCTGGAAGTTGGACACCTCAA
Il20rb	NC_000075	CGGATGCAGTGTCCGTTTTAC	GGGTTCCACATCAAGAGATGC
Socs1	NC_000082	CTGCGGCTTCTATTGGGGAC	AAAAGGCAGTCGAAGGTCTCG
Ifng	NC_000076	ATGAACGCTACACACTGCATC	CCATCCTTTTGCCAGTTCCTC
Il11	NC_000073	TGTTCTCCTAACCCGATCCCT	CAGGAAGCTGCAAAGATCCCA
Crlf1	NC_000074	CTCCCTGCAAGCTACCTGC	AGGGTGGAGGTGTTAAGGAGG
Myc	NC_000081	ATGCCCCTCAACGTGAACTTC	GTCGCAGATGAAATAGGGCTG
Pik3r1	NC_000079	ACACCACGGTTTGGACTATGG	GGCTACAGTAGTGGGCTTGG
Csf3r	NC_000070	CTGATCTTCTTGCTACTCCCCA	GGTGTAGTTCAAGTGAGGCAG
Zo-1	NC_000073	CCAGAAATACCTGACGGTGCT	AGGATGGAGTTACCCACAGC
Occludin	NC_000079	CTTACAGACCTGATGAATTCAA ACC	TGCATCTCTCCGCCATACAT
Claudin-1	NC_000082	TCAGGTCTGGCGACATTAGT	GCCAAATTCATACCTGGCATT
Claudin-2	NC_000086	CAGCTTGTGACCCCTTGGAC	GGCATCTAGAAAACGGAGCC
