# Supplementary material for: Integrative Multiomics Profiling Unveils the Protective Function of Ulinastatin against Dextran Sulfate Sodium-Induced Colitis
Source: Antioxidants (Basel). 2024 Feb 8;13(2):214. doi: 10.3390/antiox13020214 (PMC10886110; doi:10.3390/antiox13020214)
Supplement: Supplementary file 1 [file antioxidants-13-00214-s001.zip › Supplementary Table S2.pdf]

Supplementary Table S2. Antibodies used for immunohistochemistry and immunofluorescence.

Antibody	Manufacturer	Catalog	Dilution
CD4	Santa Cruz	sc-20079	1:100
CD45	Cell Signaling	#70257	1:100
Cox2	Cell Signaling	#12282	1:500
IL-1β	Cell Signaling	#12242	1:100
IL-6	Abcam	ab208113	1:50
TNF-α	Servicebio	GB11188-100	1:400
