# Supplementary material for: Integrative Multiomics Profiling Unveils the Protective Function of Ulinastatin against Dextran Sulfate Sodium-Induced Colitis
Source: Antioxidants (Basel). 2024 Feb 8;13(2):214. doi: 10.3390/antiox13020214 (PMC10886110; doi:10.3390/antiox13020214)
Supplement: Supplementary file 1 [file antioxidants-13-00214-s001.zip › Supplementary Table S3.pdf]

Supplementary Table S3. Antibodies used for western blotting.

Antibody	Manufacturer	Catalog	Dilution
GAPDH	Proteintech	60004-1-Ig	1:20000
ZO-1	Abcam	ab276131	1:1000
Occludin	Abcam	ab216327	1:1000
Claudin-1	Abcam	ab307692	1:1000
Claudin-2	Abcam	ab53032	1:1000
α-Tubulin	Proteintech	66031-1-Ig	1:20000
JAK2	Abcam	ab108596	1:1000
phospho-JAK2 (Y1007+Y1008)	Abcam	ab32101	1:1000
STAT3	Abcam	ab68153	1:1000
phospho-STAT3 (Y705)	Abcam	ab267373	1:1000
AKT	Proteintech	60203-2-Ig	1:2000
phospho-AKT (Ser473)	Proteintech	66444-1-Ig	1:2000
SOCS1	Abcam	ab280886	1:1000
