# Supplementary figures and images for: Integrative Multiomics Profiling Unveils the Protective Function of Ulinastatin against Dextran Sulfate Sodium-Induced Colitis
Source: Antioxidants (Basel). 2024 Feb 8;13(2):214. doi: 10.3390/antiox13020214 (PMC10886110; doi:10.3390/antiox13020214)

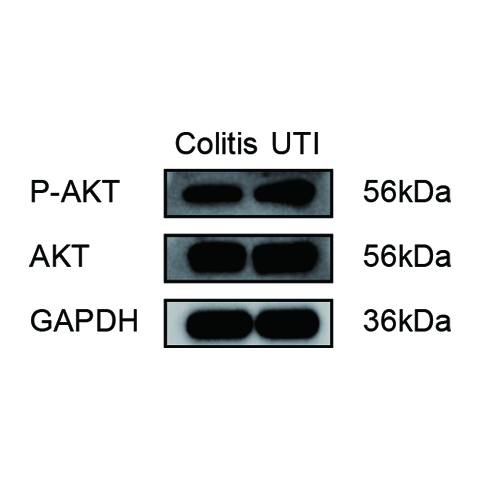

Supplement: Supplementary file 1 [file antioxidants-13-00214-s001.zip › Supplementary Figure S1.jpg]

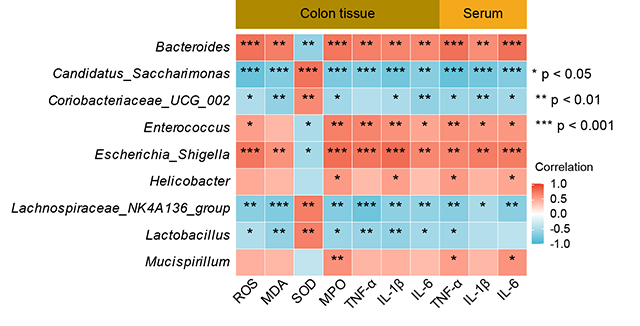

Supplement: Supplementary file 1 [file antioxidants-13-00214-s001.zip › Supplementary Figure S2.jpg]
